# A quality improvement program to improve nutritional status of children with Cystic Fibrosis aged 2-12 years old over a 3 year period at CF center Roscoff, Brittany

**DOI:** 10.1186/s13023-017-0746-6

**Published:** 2018-02-08

**Authors:** Krista Revert, Laurence Audran, Jocelyne Pengam, Pascal Lesne, Dominique Pougheon Bertrand

**Affiliations:** 1 CF center Roscoff, Fondation ildys, Roscoff, France; 2 LEPS EA3412, Sorbonne Paris Cité University, Bobigny, France

**Keywords:** Cystic Fibrosis, Quality improvement program, Therapeutic patient education, Nutritional care, Pediatric care, BMI

## Abstract

**Background:**

The Cystic Fibrosis (CF) center in Roscoff (Brittany) has been involved in therapeutic education programs (TEP) since 2006 and took part in the pilot phase of the French quality improvement program (QIP) since 2011. The aim was to improve the nutritional status of children with cystic fibrosis aged 2-12 years old in order to optimize their health status as they enter adolescence.

**Methods:**

A multidisciplinary quality team was created in order to select and address a specific health problem among our pediatric population. Following analysis of yearly indicators for our CF center, our team chose to improve quality of care concerning nutritional status of children aged 2-12 years old. Factors influencing efficacy were studied, tools were developed to implement a new nutritional program, results were analyzed on a real-time basis.

**Results:**

Over the 3 year period, all patients from 2 years of age, were monitored with the new follow-up program (2012: *N* = 34; 2014: *N* = 44). Each patient was followed up at every clinic visit, their BMI z-score was calculated to decide their nutritional risk and personalize their follow-up program consequently. Between 1/1/2012 and 31/12/2014, the mean BMI z-score of the open cohort improved from −0.49 to −0.22.

**Conclusions:**

Since 2014, focus on nutrition using the newly-adapted program has become routine practice at each follow-up visit. Patients and parents expressed a high level of satisfaction (75% very satisfied). The follow-up program aimed at improving nutritional status for children aged 2-12 years old was successfully implemented and integrated into routine practice; it was therefore extended to all children with CF (1 month - 18 years) in our center. The relationship among professional and patients and parents was strengthened.

## Background

The prognosis of Cystic Fibrosis (CF) patients is mainly related to their respiratory status. It is therefore vital to maintain the best possible respiratory function over time and especially during childhood to permit normal lung growth [1].

The direct relationship between nutritional status at the age of 2 years and FEV1 at 6 years is well established among children with CF [2].

In France, children have been followed - up for CF in specialized centers following newborn screening as of 2002. Systematic newborn screening exists in Brittany (region with the highest prevalence rate of CF in France) since 1989. Our patient cohort of 142 patients includes 70 children <18 years old. Children are first seen at our center at the age of 1 month for diagnosis. Follow-up visits are then programmed regularly with experienced professionals.

**Fig. 1:**
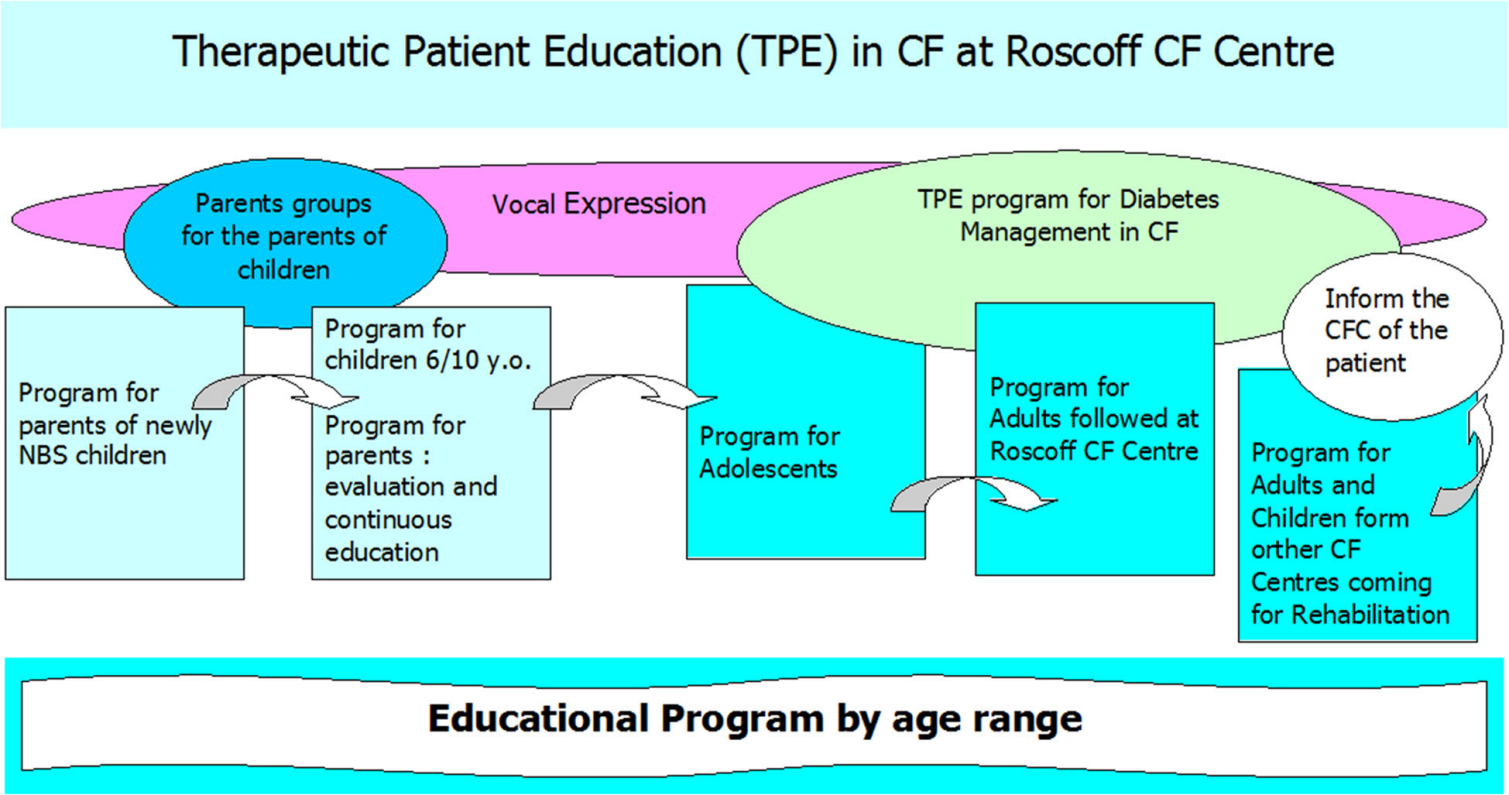
Patient Education program at Roscoff CF Centre

Therapeutic patient education (TPE) as defined by WHO in 1985 [3] as “helping patients and their parents to acquire or maintain the competencies they need to manage as well as possible their lives with a chronic disease” is implemented in our CF center since 2006 and programs have been specifically designed for parents of young children (1 month-5 years old), for children from 6 to 10 years old and their parents and also for adolescents (11-16 years old) (Fig. 1).

Our CF center Roscoff participated in the pilot phase of the QIP PHARE-M *(Programme Hospitalier d’Amélioration des Résultats et de l’Expertise en Mucoviscidose – A hospital-based program for improvement of results and expertise in cystic fibrosis care)* [4] in 2011-2012. Following initial training, review of our 2010 data showed a BMI z-score average of −0,49 for patients aged 2-12 years old. The French CF registry had data for children <18 years old but no data for the group 2-12 years old. The national median BMI z-score for <18 years in 2010 was −0.35, and in our center for the same age group was −0.5 [5]. Our multidisciplinary team chose to address the nutritional status of 2-12 year-old CF patients as our results for this age group showed a very large variation in BMI z-score with a mean value of −0.49 (range: −3.5 z-score to +1.8 z- score). This significant variability in our values for this group of children thus left room for improvement. All children aged 2-12 years (34 patients) followed-up at our center were included in the program.

Our aim was to attain an average BMI z-score = 0 by end of 2014. We also expected an impact on FEV1 for patients (> 5 years old) at the end of the program in 12/2014.

## Methods

A quality team comprising a pediatrician, a dietitian, an adult patient, a social worker, a physiotherapist, a nurse coordinator and a study-coordinator was established. The team participated in 4 training sessions organized by the National training-team for CF centers participating in PHARE-M pilot phase. The pediatrician also had the opportunity to participate in a similar Learning and Leadership Collaborative face-to-face meeting in the USA organized by the CF Foundation in Anaheim (2011).

Following these training sessions, our team evaluated nutritional indicators in our center based on annual data and analysis of patient records: BMI z-score, number of clinic and dietitian visits/year, number of stool fat analysis/year, number of nutritional supplements prescribed.

**Fig. 2:**
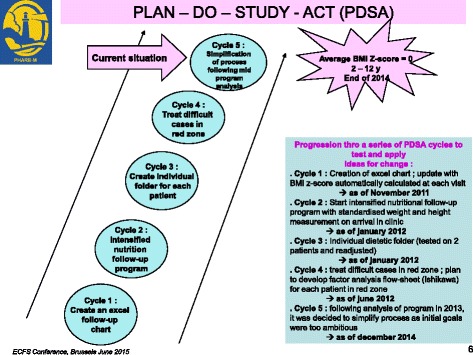
PDSA Cycles at Roscoff CF centre

We used a tool, the Ishikawa fishbone cause and effect diagram [6], to determine positive and negative factors influencing nutritional status among our patients. The main factors involving *patient and family* identified by the team were: insufficient knowledge concerning nutrition and link with respiratory status, how to titrate pancreatic enzymes according to fat intake and symptoms, reluctance to do stool sampling and fear of nasogastric (NG) tube feeding. For professionals, we noted the same reluctance to talk about NG tube feeding, difficulty in obtaining up-to-date information on weight gain or loss between clinic visits and need for more training on patient therapeutic education. The team then reflected on ideas for change and applied the PLAN-DO-STUDY-ACT (PDSA) cycle to design, implement and evaluate new tools (Fig. 2):

PDSA1: Creation of an Excel flow chart to follow up each patient over 3 years with calculation of BMI z-score classified in color categories reflecting the “at risk” nutritional status of the child: red for severe risk (BMI z-score < -1,5); orange for moderate risk (-1,5 < BMI z-score < -0,5); yellow for mild risk (-0,5 < BMI z-score < 0); green for no risk (=/>0 BMI z-score).

**Fig. 3:**
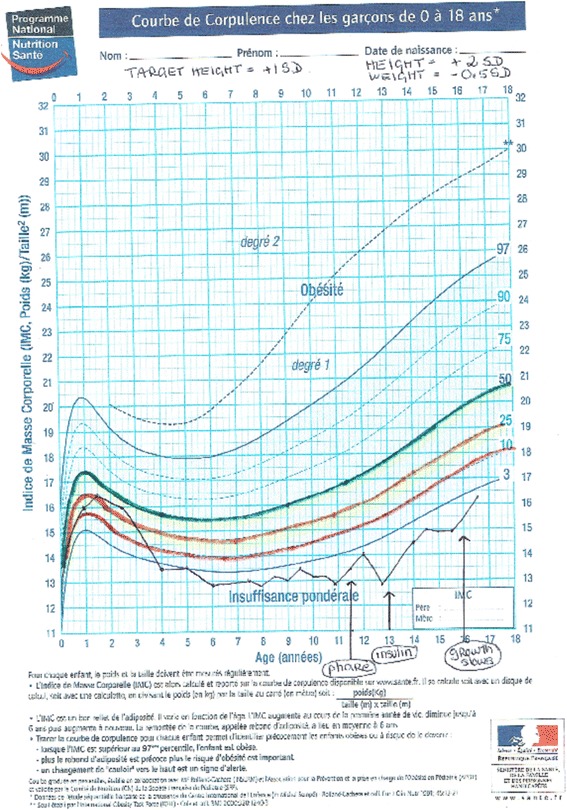
Examples of BMI color-zones on Health Ministry BMI curves for a Boy

**Fig. 4:**
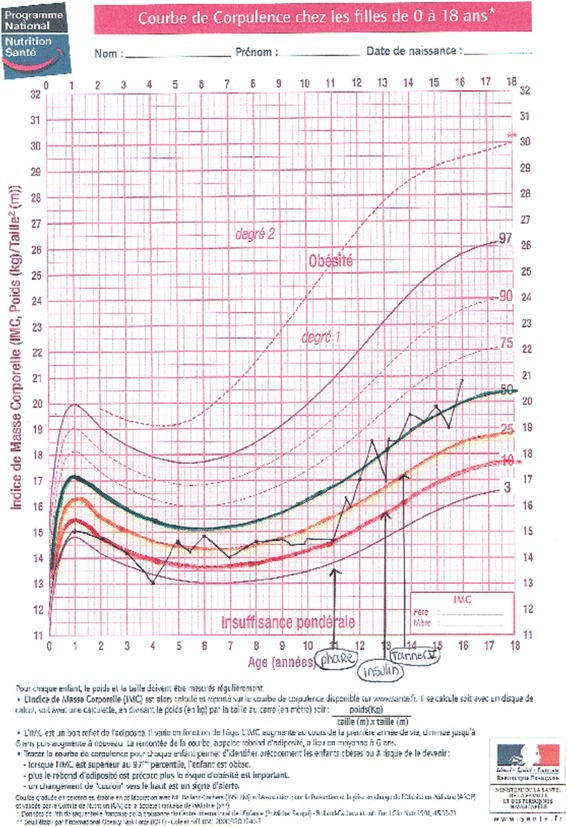
Examples of BMI color-zones on Health Ministry BMI curves for a Girl

**Fig. 5:**
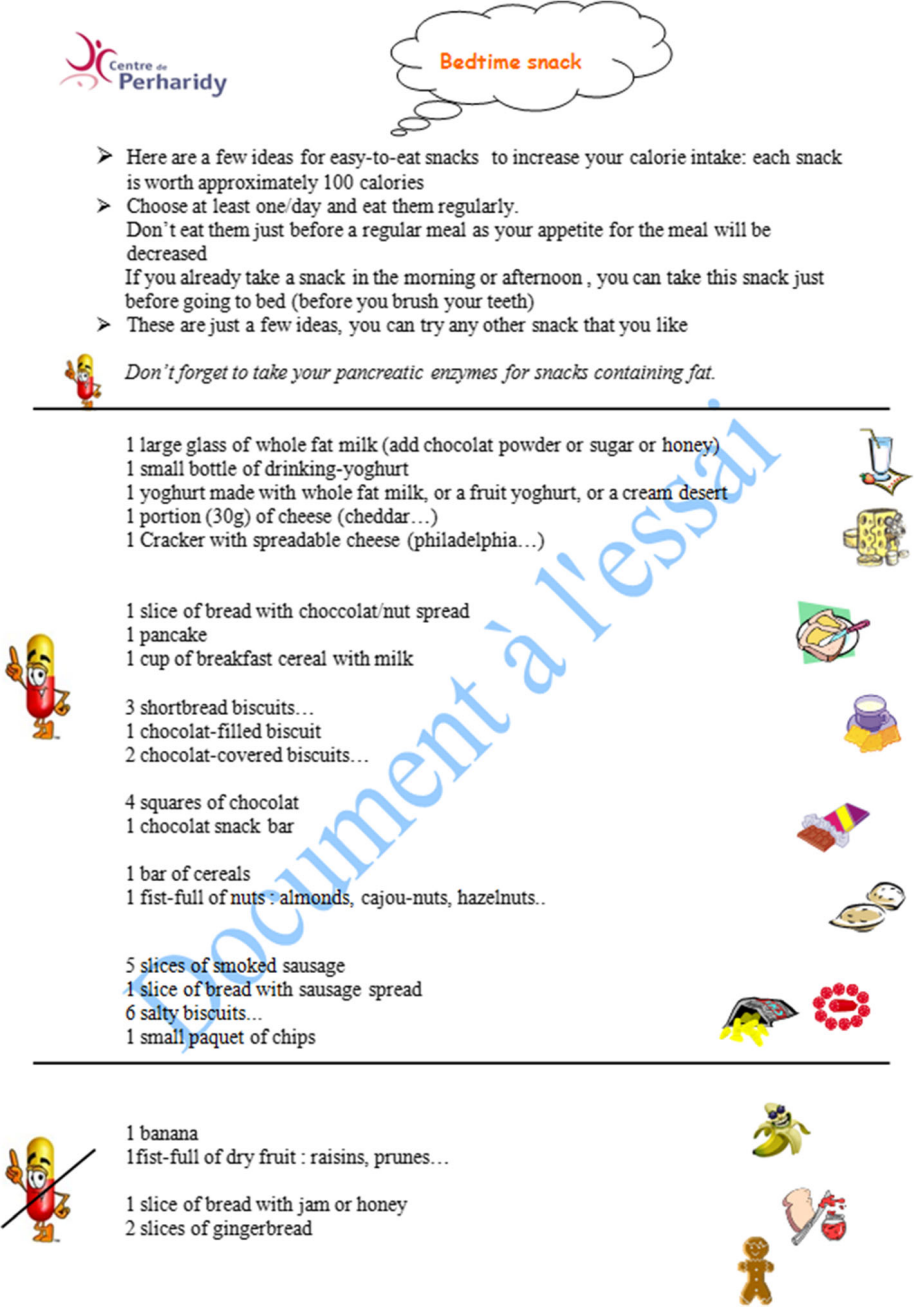
100-calorie Snacks for children

PDSA2: Creation of a personalized folder for each patient comprising: a simplified explication of the link between nutritional and respiratory status according to Yen et al. [2] publication highlighting the close correlation between a good nutritional status at 2 y and subsequent pulmonary function; a color-coded BMI chart to be updated at every visit using national BMI curves for girls and boys from Ministry of health [7] on which the 4 colors were added to correlate with the selected at-risk categories on the Excel flow chart (Figs. 3 and 4); a list of ideas for 100-calorie-snacks (Fig. 5) illustrated by the dietitian; Individualized weight gain goals with home weighing sheet for orange and red groups

**Fig. 6:**
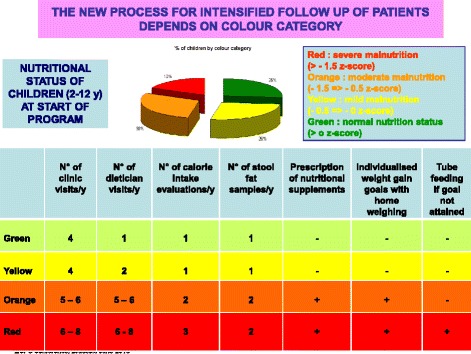
Initial intensified follow-up by color category

PDSA3: Intensification of our follow up program according to the child’s BMI color category including number of clinic visits, dietitian visits, calorie-intake evaluations, stool fat analysis, prescription of nutritional supplements (Fig. 6).

Therapeutic patient education (individually adapted) was proposed to all patients and parents according to their needs and age group: for the 0-4 year-old group, the program for parents was finalized in 2011; for the 6-10 year-old group, the program for children and parents was created and implemented during the study period; for the 10-16 year-old group, the program has been implemented since 2010.

Difficult cases in red zone were specifically reviewed at multidisciplinary staff meetings for analysis of individual causal factors (positive and negative) and discussion of the next step to be implemented. For all patients, psycho-social support by both team psychologist and social worker was offered and early discussion concerning NG tube feeding took place systematically with all families.

Satisfaction among patients and parents was assessed using a paper survey given to the patient/parent at a clinic visit (75% responded).

A visual display area (poster) was set-up in the out-patient and in–patient departments so that all the patients, families and professionals could be kept up to date on progress.

## Results

All pediatric patients aged 2-12 years old followed up at our centre (34 patients) were enrolled in January 2012. Each child coming to a clinic visit at or after their second birthday was subsequently enrolled. All children were kept in the program for 3 years even after their 12th birthday; therefore the cohort increased to a total of 44 patients by December 2014. One child was excluded after 1 year as he was accepted on lung and liver transplant list in another centre. All except one patient had pancreatic insufficiency, 44% were girls, 88% were diagnosed by new-born screening. Eighteen percent were colonized by Pseudomonas aeruginosa, 9% by MRSA (Methicillin resistant *Staphylococcus aureus*) and 9% by Burkholderia cenocepacia or Inquilinus limosus.

### Impact on patient health outcomes

The mean BMI z-score of the open cohort of children (34 at the start and 44 at the end of the program) progressed from −0.49 (SD = 0,89) in December 2011 (just before starting the program) to −0.22 (SD = 0,97) in December 2014. Comparison of our entire pediatric group of patients (0-18 years) with the national median showed a progression in median BMI z-score for our center from −0.5 to −0.26 z-score over the 3 years whereas the national figures progressed from −0.32 to −0.28 z-score [5].

**Fig. 7:**
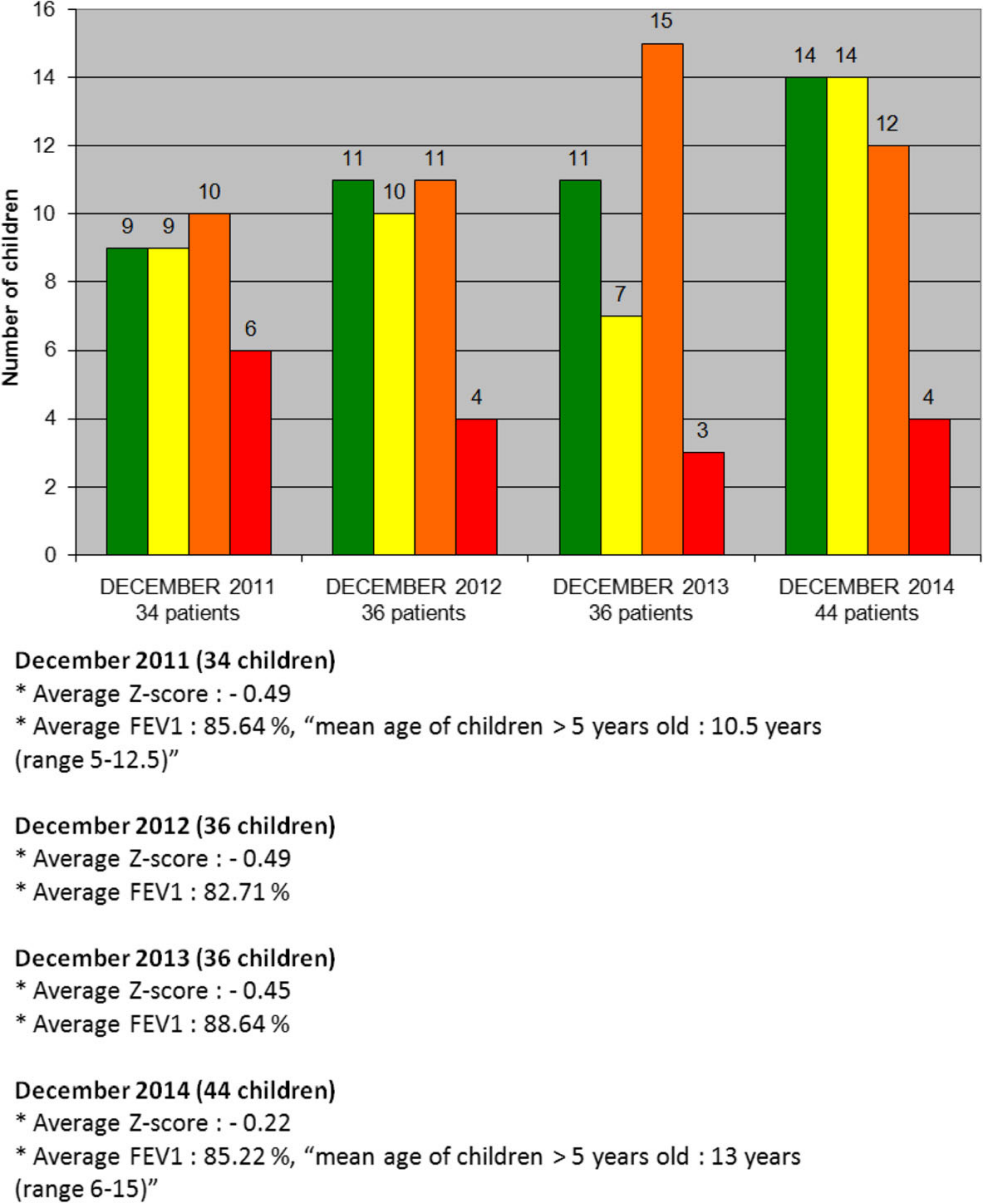
Number of patients, mean BMI z-score and FEV1% by color category

The progression is also shown in the percentage and number of patients in each color category over the 3 years (Fig. 7). Moreover, average FEV1 for children >5 years showed no decline through this 3 year period at 85,5% despite increasing age of the cohort (mean age at the start of the program: 10,5 and 13 at the end of the program). (Fig. 7 Continued).

### Impact on the process of care

**Fig. 8:**
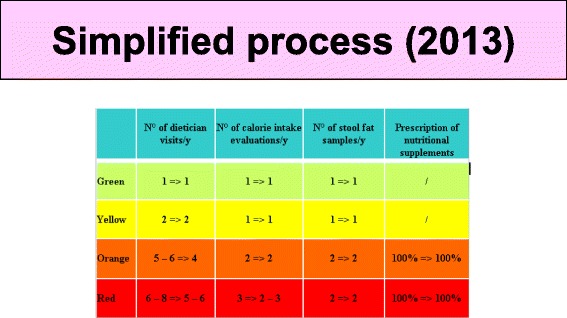
Simplified follow-up process by color category after 2013

This new follow-up program included increased number of clinic visits for patients in red and orange zones. These were difficult to achieve as our center is in a rural area making transportation a limiting factor. For this reason, the program was adjusted and simplified in 2013 with fewer clinic and dietitian visits and increased telephone contacts (Fig. 8).

Seven patients who were not improving their BMI z-score despite close follow-up – two were stable, five were deteriorating – were screened for other diagnoses related to nutritional status such as diabetes, coeliac disease and helicobacter infection [8, 9]. Early-stage diabetes was detected and treated for 3 patients, one patient was treated for helicobacter infection and one patient had supplementary investigations for suspected coeliac disease (not confirmed on biopsy). These screening tests are now part of our routine check-up.

### Impact on the team

The project was well received by all the professionals involved. For the first 18 months, the pilot team met very regularly to plan and discuss progress, prepare results and presentations. Over the following 18 months, meetings were more focused with often just 2 or 3 members (dietitian, pediatrician and study coordinator). The study coordinator entered all the data from the patient clinic visit on a real time basis so results were available at each meeting.

The multidisciplinary team received training on patient therapeutic education at a national training Institute. The majority had already received training prior to the program, the others received training throughout the program.

Patients in red zone were presented more frequently at the weekly multidisciplinary staff meetings for input by all members. Outcome of discussions was entered into their files.

The quality improvement program was presented once to the hospital management/administration, twice to the multidisciplinary team, and was selected as a subject for examination by the external health authorities audit team as an example of our hospital’s improved quality of care.

### Other benefits for patients

Patients and parents were very involved in the program and motivated to improve their position on the colored BMI curve. The patient therapeutic education program (6-10y) developed during this period was rapidly applied and was a support to the program.

The process, program and results were displayed in both out-patient and In-patient Departments so all patients and parents had a simple visual summary of the program with update on results.

Satisfaction among patients and parents was assessed using a paper survey given to the patient/parent at a clinic visit (75% responded): results showed that 75% were very satisfied overall especially concerning individual folder with calorie sheet (70%), information given about the program (66%), and concerning intensified follow-up of children in orange/red zones (48% very satisfied, 46% moderately satisfied).

### Inspiration for other CF centers

The presentation of our work at French PHARE-M training sessions and post training sessions allowed us to share our tools, process of care and results with the other teams involved in the QI program. The tools were put on the PHARE-M website and were used by teams in other centers wishing to improve their patient BMI z-score.

We took the opportunity to present our work at 2 ECFS Conferences and were selected in 2012 and 2015 for the poster session. Moreover, the pediatrician was invited to present the program at the European quality management training course held in 2015 and 2016 in the form of video sequences to illustrate the steps of the method: 5 point analysis - selection of global aim – PDSA cycles and results .

## Discussion

CF center Roscoff succeeded in improving the nutritional status of young children with CF thereby also maintaining good respiratory function and thus giving them a better start into adolescent and adult life. We did not achieve our initial target (median: 0 z-score) but did improve the nutritional status over the 3 year period. Comparison of our entire pediatric group of patients (0-18 years) with the national median showed a progression in median BMI z-score for our center from −0.5 to −0.26 z-score over the 3 years whereas the national figures progressed from −0.32 to −0.28 z-score [5]. Statistical analysis was not carried out as the cohort was open and numbers insufficient.

Patient education played an important role in the program allowing parents and children to acquire skills and autonomy. Intensification of follow-up according to the “at risk” status of the child was instrumental and systematic screening for coeliac disease, early diabetes and helicobacter infection was implemented to identify individual causes of poor weight gain. The dietitian’s involvement was a key-role as her time was increased in order to see more children at clinic visits, to analyze calorie-intake, carry out education sessions, coordinate with the multidisciplinary team, enter data according to color zone and design new educational tools. The cohesion of the team around the physician leader ensured consistency of actions and was even enhanced throughout the project.

C. McDonald [10] describes a similar nutritional risk screening tool for 2-20 year old patients with CF, based on weight and height velocities using an algorithm to attribute points which then determine risk. Color codes were also used for patient and parent motivation. On publication no data were available to determine impact on patient outcome.

Our experience with 2-12 year-old patients and their families show that nutritional outcome can be optimized through close follow-up including patient and family education along a “pathway” during childhood [11]. In CF, nutritional status is dependent on pancreatic enzyme adherence at home and on learning how to titrate the dose according to fat intake and symptoms [9]. Training for the staff was useful to foster the importance of patient education. Pulmonary function of patients older than 5 showed no decline during the 3 year follow-up despite increasing age of the cohort which favors a better prognosis for their adolescence and adult life (a decline in FEV1 of 1,4% per year was described by Welsh et al. increasing to 2,6% per year during adolescence) [12].

### BMI is not the sole indicator of good nutritional status

The cohort was heterogeneous including for example “tall thin family-pattern” children who had excellent growth in height following a curve at +2 or +3 SD with good weight gain following a median curve, good bone and lean body mass index but had however a BMI in the orange/red zone. The data for these patients explains the wide range of SD in our final results. In fact, only once these children’s growth in height flattened off at the end of puberty did we see an improvement in BMI z-score (example Fig. 3). This is one of the reasons explaining why we did not attain BMI z-score = 0 at the end of the study as 2 “tall-thin” patients stayed in the red zone throughout the study period.

### Adjusting doses of pancreatic enzymes

For 24 children receiving relatively high doses of pancreatic enzymes (>10,000 U/kg) but still in orange or red zones or presenting signs and symptoms of persistent fat-malabsorption, we combined use of 2 different pancreatic enzymes, active at different PH s (5,5 and 7) thus at different zones in the gastrointestinal tract, without increasing the total dose in order to maximize fat absorption. Our hypothesis is that it is probable that not all patients achieve a PH at 7 in the duodenum due to dysfunctional bile salt secretion in CF. For 46% of patients for whom a mix of the 2 types of pancreatic enzymes was prescribed, we noted a substantial improvement in BMI z-score (average + 0.7) within the following 12 months. This impact could lead to a further research study.

### Prospects

The program continues after 2014 as new techniques and new-change ideas continue to be implemented.

Performing continuous glucose monitoring led to early intervention with insulin therapy (0,25 U/kg of long acting insulin) following diagnosis of significant glucose intolerance or early diabetes (Fig. 4). This monitoring was greatly facilitated by use of the FREESTYLE device as children did not have to do any finger-prick controls.

Children’s technique for spirometry test was often quite deficient with inconsistent results depending on the child’s motivation that day. For this reason a specific module was created in the patient therapeutic program to prepare 5 year old children for the first test with the physiotherapist assisting at the examination to ensure the best possible technique. Subsequently lung function evaluation included LCI (lung clearance index) performed yearly as this test is much less dependent on technique/motivation to obtain realistic results.

### Benefits for the quality team

The team followed the framework proposed by PHARE-M; there was good cohesion and implementation as all professionals were kept up to date in the program. The follow-up indicators were updated at each visit on a real-time excel chart which motivated all actors to encourage the best possible results for their patients. The team experienced some difficulties in maintaining regular meetings which were sometimes replaced by smaller more focused discussions.

## Conclusion

We have demonstrated that the program is easily integrated into normal clinical practice and has been extended to all pediatric patients (1 month - 18 years old) as of 1/2015. This patient – centered process including individual patient therapeutic education and individual goals helped maintain the dynamic of care which continues up to now.
